# Biotechnological Methods for Buckwheat Breeding

**DOI:** 10.3390/plants10081547

**Published:** 2021-07-28

**Authors:** Zlata Luthar, Primož Fabjan, Katja Mlinarič

**Affiliations:** 1 Biotechnical Faculty, University of Ljubljana, SI-1000 Ljubljana, Slovenia; pf2253@student.uni-lj.si; 2 Gregorčičeva 4, SI-9252 Radenci, Slovenia; katjamlinaric@hotmail.com

**Keywords:** transcriptomics, genomics, interspecific hybridization, tissue cultures, molecular markers, metabolites

## Abstract

The *Fagopyrum* genus includes two cultivated species, namely common buckwheat (*F. esculentum* Moench) and Tartary buckwheat (*F. tataricum* Gaertn.), and more than 25 wild buckwheat species. The goal of breeders is to improve the properties of cultivated buckwheat with methods of classical breeding, with the support of biotechnological methods or a combination of both. In this paper, we reviewed the possibility to use transcriptomics, genomics, interspecific hybridization, tissue cultures and plant regeneration, molecular markers, genetic transformation, and genome editing to aid in both the breeding of buckwheat and in the identification and production of metabolites important for preserving human health. The key problems in buckwheat breeding are the unknown mode of inheritance of most traits, associated with crop yield and the synthesis of medicinal compounds, low seed yield, shedding of seeds, differential flowering and seed set on branches, and unknown action of genes responsible for the synthesis of buckwheat metabolites of pharmaceutical and medicinal interest.

## 1. Introduction

*Fagopyrum* is a small genus that includes two cultivated species and more than 25 wild buckwheat species, mostly endemic to southwest China, which is considered to be the origin, distribution, and diversity center [1,2,3,4]. Two species, common buckwheat (*F. esculentum* Moench) and Tartary buckwheat (*F. tataricum* Gaertn.), are cultivated and spread throughout the world. Both species have benefits to human health and are gluten-free [5]. Of all cereal foods, these species have the most balanced content of amino acids necessary for humans [6,7,8,9]. Some nutrients, such as starch and protein, can bind to phenols in complexes, which can slow digestion [10,11,12,13]. They contain more dietary fiber than other cereals and are rich in flavonoids [14,15,16,17,18,19]. The genetic background of the synthesis of buckwheat flavonoids was studied by Zhou et al. [20]. Buckwheat can be a nutritional source of fatty acids and mineral elements, including selenium and iodine [21,22,23,24]. Flavonoids, other phenolic substances, and selenium are involved in the protection of buckwheat plants from UV-B radiation [25,26,27]. The wild species *F. cymosum* (Meisn.) is widespread in Southern Asia and is utilized as forage and a source of pharmaceutical drugs [28,29]. Other wild species within the genus *Fagopyrum* are not important for agricultural production, but they can be an important source of genes for breeding. Therefore, their discovery in nature and systematic studies is very important.

Molecular systematic research and genome studies in the last two decades have discovered new species and their proper classification into the genus *Fagopyrum* (Table 1) [2,4,28,30,31,32,33,34,35,36,37,38,39,40,41,42,43].

Buckwheat breeding is focused on seed yield and the quality of cultivated common and Tartary buckwheat. Cultivated species and varieties are annual. At present, the average yields of common and Tartary buckwheat are about 1050 and 1800 kg/ha (with the highest yield of 2500 and 3000 kg/ha), respectively. Allnatural perennial buckwheat species are wild types. They have many negative traits, including seed shattering, high sensitivity to the duration of light and dark (photoperiods) and temperature, indeterminate flower and fruit development, a non-compact plant, low fertility and yield, strong seed dormancy, and uneven germination, etc. [44].

The goal of breeders is to improve these properties with existing methods of classical breeding, with biotechnological processes or a combination of both. Future work will include the use of transcriptomics and genomics to aid in both the breeding of and in the identification and production of medicinal compounds in buckwheat. At present, there remain few key problems in the buckwheat breeding field: (i) unknown mode of inheritance of most traits, associated with the crop and synthesis of medicinal compounds; (ii) low seed yield; (iii) shedding of seeds; (iv) sensitivity to low temperatures; (v) differential flowering and seed set of branches; and (vi) perennial habit buckwheat, etc.

## 2. Species of the Genus *Fagopyrum* as a Possible Source of Germplasm for Breeding and Their Reproductive Properties

**Figure 1:**
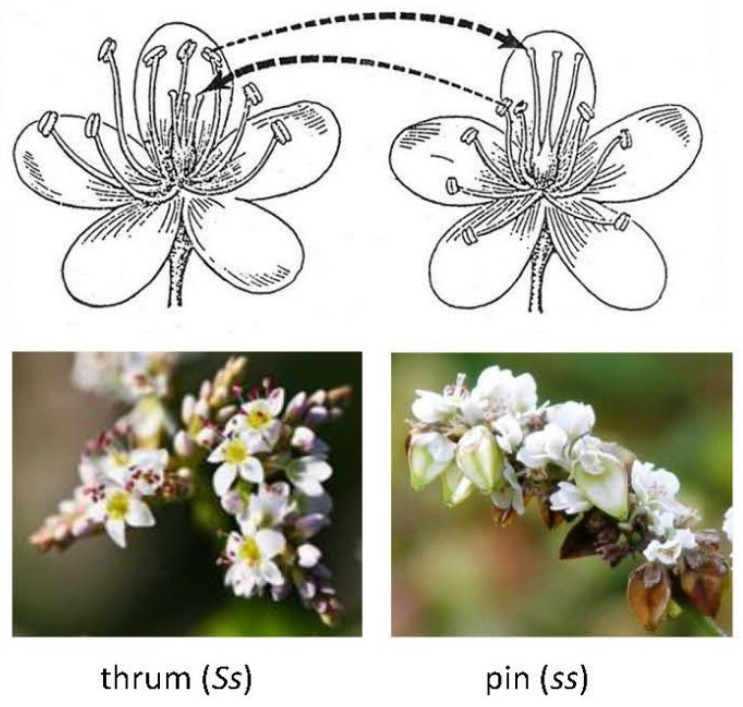
Morphology of flowers and cross-pollination between pin and thrum plants of *Fagopyrum esculentum*: left—thrum (*Ss*), short-styled and long anther and right—pin (*ss*), long-styled and short anther. The arrows show compatible cross-pollination.

Within the genus *Fagopyrum*, there are self-pollinating and cross-pollinating species with three types of flowers: short three-parted styles and long anthers (thrum type, *Ss*), long-styled and short anthers (pin type, *ss*) within the species (*F. esculentum*, *F. cymosum*, etc.) (Figure 1), and long homo-styled flowers (LH type) (*F. tataricum*, *F. homotropicum*, etc.) (Table 1). Thrum and pin flowers within a species represent one hetero-styled group with a self-incompatible pollination mechanism, and long homo-styled flowers represent another group with hetero-styled flowers with self-compatibility.

Molecular systematic studies using multiple accessions of the species and different markers on nuclear and plastids genomes are needed to clarify the evolutionary position of the species [45,46,47,48,49]. Ohnishi and Matsuoka [50] applied isozyme markers and RFLP of chloroplast DNA (cpDNA) to a phylogenetic study of the genus *Fagopyrum*. Both studies confirmed that the genus is divided into two major groups, one including two cultivated species *F. esculentum* and *F. tataricum* and their wild relative *F. cymosum* and the other composed of other wild species. Molecular systematic studies of species by different markers, mainly on the nuclear genome, are needed to clarify the evolutionary position of the species.

Among the *Fagopyrum* genus currently accepted species, there are 16 hetero-styled self-incompatible species, 6 hetero-styled self-compatible species and 7 homo-styled self-compatible species; in one species (*F. tibeticum*), the pollination system is not exactly known.

The basic chromosome number of *Fagopyrum* species is n = 8. Most species are diploid with 2n = 16. *F. pilus, F. megaspartanium,* *F. hailuogouense,* *F. gracilipes, F. crispatifolium*, and *F. rubifolium* are tetraploid with 4n = 32. *F. esculentum, F. cymosum*, and *F. homotropicum* have both diploid and tetraploid forms [51]. *F. tibeticum* has a chromosome number 2n = 48 (Table 1) [2,3,52].

**Table 1:** *Fagopyrum* species: hetero-styled self-incompatibility (SI), hetero-styled, self-compatibility (SC) and homo-styled self-compatibility (SC), ploidy level and genome size.

Species	Nuclear DNA Amount (pg) *	C-Value **	Ploidy/Chromosome Number	References
Mating system: hetero-styled species, thrum/pin flower, self-incompatibility (SI)				
*F. esculentum*	2.77 5.49	1.39 1.37	2n/16 4n/32	[30,53,54,55,56]
*F. cymosum*	2.32 3.37	1.16 0.48	2n/16 4n/32	[54,55,57]
*F. lineare*	1.08	/	2n/16	[58,59]
*F. urophyllum*	3.83	/	2n/16	[60,61]
*F. statice*	1.35	0.68	2n 16	[61]
*F. leptopodum*	1.43	0.72	2n/16	[62,63]
*F. gilessii*	1.80	/	2n 16	[63]
*F. capillatum*	1.71	0,68	2n/16	[30]
*F. gracilipedoides*	/	/	2n/16	[34]
*F. jinshaense*	/	/	2n/16	[34]
*F. pilus*	1.52	/	4n/32	[33]
*F. megaspartanium*	/	/	4n/32	[33]
*F. densovillosum*	/	/	2n/16	[64]
*F. qiangcai*	/	/	2n/16	[38]
*F.luojishanense*	/	/	2n/16	[39]
*F. hailuogouense*	/	/	4n/32	[40]
Mating system: hetero-styled species, self-compatibility (SC)				
*F. pleioramosum*	3.05	1.53	2n/16	[30]
*F. callianthum*	/	/	2n/16	[30]
*F. macrocarpum*	2.32	/	2n/16	[32]
*F. wenchuanense*	/	/	2n/16	[38]
*F. longzhoushanense*	/	/	2n/16	[42]
*F. longistylum*	/	/	2n/16	[4]
Mating system: homo-styled species, self-compatibility (SC)				
*F. tataricum*	1.11	0.56	2n/16	[53,65,66]
*F. homotropicum*	2.46 5.20	1.23 1.30	2n/16 4n/32	[30,66]
*F. gracilipes*	3.35	0.84	4n/32	[62,67]
*F. crispatifolium*	/	/	4n/32	[35]
*F. pugense*	/	/	2n/16	[36,39,41]
*F. rubifolium*	3.31	/	4n/32	[32]
*F. zuogongense*	/	/	2n/16	[33]
Mating system: unknown				
*F. tibeticum*	/	/	2n/48	[68]
Interspecific hybrid *F. cymosum* × another species with hetero-styled flowers				
*F. giganteum*	2.52	/		[69]

* [70] Nagano et al., 2000, ** genome size of the haploid nucleus, /—unknown.

## 3. Heteromorphic Self-Incompatibility in Buckwheat and Mode of Inheritance

Common buckwheat (*Fagopyrum esculentum*) is a highly allogamous crop due to the existence of peculiar dimorphic and sporophytic types of self-incompatibility, a heteromorphic strictly self-incompatible (SI) crop. The flowers are perfect but incomplete. There are no petals, but the calyx is composed of five petal-like sepals. This species has plants bearing one of the two floral types: one with long styles and short stamens, with styles that project 0.5–2 mm over the anthers (so-called pin flowers) and one with short styles and long stamens, with styles that reach about the level of the middle of the filaments of the anthers (so-called thrum flowers). One plant can only have one type of flower, and the same type of flower cannot pollinate another, which is why it needs insects for cross-pollination between the pin and thrum plants, which makes it difficult to breed homozygous and homogenous pure line varieties and for economic traits (Figure 1 and Table 1) [41,48,71,72,73,74,75].

Heteromorphic self-incompatibility in common buckwheat is controlled by a single gene locus (*S* locus), sometimes also called the *S* supergene, and segregates as a simple Mendelian character [76,77]. Pin plants are homozygous for the *S* locus (*ss*), and thrum plants are heterozygous (*Ss*) [78,79].

The *S* locus controlling heteromorphism of the distylous species is closely related to the loci controlling sporophytic incompatibility reactions. Sharma and Boyes [80] postulated that the supergene *S* is composed of five genes: *G*—style length, *I^S^*—stylar incompatibility, *I^P^*—pollen incompatibility, *P*—pollen size, and *A*—anther height or filament length. Gene *I^S^* is tightly linked or pleiotropic with *G*, and *I^P^* is tightly linked or pleiotropic with *A*. Common buckwheat is therefore strictly self-incompatible. The genetic model represents a thrum genotype as *Ss* (*GI^S^I^P^A*/*gi^S^i^P^pa*) and a pin genotype as *ss* (*gi^S^i^P^pa*/*gi^S^i^P^pa*). This implies that a 1:1 ratio between the two morphs is conserved from generation to generation.

Yasui et al. [81] performed a transcriptome analysis of the genes expressed in the styles of pin and thrum plants. They identified a gene that is expressed only in the short-style plants and is completely linked to the S locus. Identified gene was a homolog of *Arabidopsis thaliana* ELF3 and was therefore named *S-LOCUS EARLY FLOWERING 3* (*S-ELF3*).

## 4. Interspecific Hybridization

Some self-compatible (SC) lines have already been developed by the interspecific crossing of *F. esculentum* and *F. homotropicum* [82,83,84]. The allele controlling the homomorphic flower type and self-compatibility was named *S^h^* and was derived from recombination in the supergene *S. S^h^* is dominant over the *s* allele and recessive over the *S* allele. Therefore, at a first locus is located the supergene *S* with three alleles and their intrallelic interaction *S* > *S^h^* > *s* [83]. At a second locus is located a diallelic *S^c^* gene responsible for the morphology of the reproductive organs. The two complementary dominant genes *S^h^* and *S^c^* control self-compatibility in *F. homotropicum* [28,77].

SC lines have a potential for high and stable yields that do not need to rely on pollinators, and their useful agronomical traits can be easily fixed [85]. However, plants obtained by interspecific crossing have undesirable traits such as lodging and shattering, which can be removed by repeated backcrossing with elite buckwheat varieties. More such crosses are needed to produce SC varieties with desirable traits for farmers and consumers [85,86].

The maps of the *S^h^* allele in buckwheat by random amplified length polymorphism (RAPD) and amplified fragment length polymorphism (AFLP) markers are presently available [82,87,88]. Following incompatible self-pollination (intra-morph pollination), pollen tube growth is inhibited at two-third of the style length in the long styles of pin flowers and at the junction between the stigma and the style in the short styles of thrum flowers [89]. In consequence, pollen tubes stop at nearly the same distance from the ovary in both morphs. Miljus-Djukic et al. [90,91] detected some proteins in the styles 2 h after compatible and incompatible pollinations. The proteins responsible for the self-incompatibility response are not the same in the long and the short styles. These authors assume that some of the proteins probably have a role in pollen adhesion to the stigma surface and in the inhibition of the pollen tube elongation.

As mentioned above, the *S^h^* allele, conferring the SC, is dominant over the *s* allele but recessive to the *S* allele. To obtain F1 plants that are self-compatible, SC plants (*S^h^*/*S^h^)* and pin plants (*s*/*s*) need to be crossed. In the subsequent generations (F2 and so on), it is easy to determine whether a plant is SC or SI from flower morphology, but we cannot distinguish the *S^h^*/*S^h^* and *S^h^*/*s* genotypes based on the flower morphology. Heterozygous plants (*S^h^*/*s*) would segregate SC and SI plants in the next generation. To accelerate the development of crosses from SC lines and elite varieties, and buckwheat breeding in general, codominant markers that will be able to distinguish homo- and hetero-zygosity at the *S* locus will have to be developed [85].

**Figure 2:**
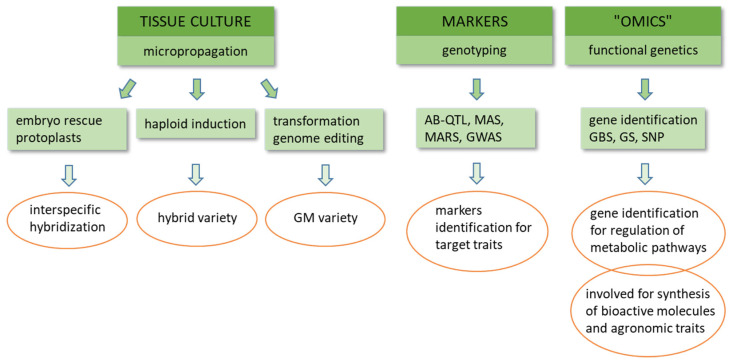
Integrative genomics and breeding approaches for accelerated genetic improvement of buckwheat: GM—genetically modified, AB-QTL—advanced backcross quantitative trait locus, MAS—marker assisted selection, MARS—marker assisted recurrent selection, GWAS—genome wide association study, OMICS—refers to a field in biological sciences, such as genomics, transcriptomics, proteomics, and metabolomics, GBS—genotyping by sequencing, GS—genomic selection, SNP—single nucleotide polymorphism.

Matsui et al. [85] used next-generation sequencing (NGS) analysis in combination with bulked-segregate analysis to develop codominant STS DNA markers that are able to distinguish between *S^h^*/*S^h^* and *S^h^*/*s* plants. They developed 4 codominant markers that are tightly linked (one was less than 0.1 cM from the *S* locus) (Figure 2). Three of the four markers showed a high level of polymorphism between the SC line and breeding lines in Japan, and the authors concluded that these three markers would be useful for introducing SC through the *S^h^* allele into many buckwheat varieties.

Chen [92] started to carry out perennial buckwheat breeding, including the perennial buckwheat, by selective breeding, cross breeding, and interspecific hybridization breeding. One way to improve perennial wild buckwheat is by selective breeding. There are many mutations that have occurred in natural populations of perennial buckwheat. In order to develop many varieties with a high flavonoid content in the leaves and flower of the *F. cymosum* complex, 360 accessions were collected nationwide in China, and their flavonoid content in leaves and flowers of species including *F. megaspartanium*, *F. pilus*, and *F. cymosum* was estimated. The first perennial variety from natural perennial species, ‘Gui Jinqiaomai No.1’, was developed in 2005. It is from diploid (2x = 16) perennial *F. megaspartanium* populations native to Guiyang by single selection. Because it has high flavonoid content of up to 10.2% in leaves, much higher than the average 4.5%, it was used for harvesting leaves and making leaf tea products. Seed harvest is difficult due to shattering; therefore, it is reproduced via cutting from branches. Adaptation is difficult due to its high sensitivity to temperature, light intensity, and length of the day, which limits its range.

Interspecific hybridization has proved to be very difficult for producing normal hybrid seeds. The reason for this may be the lack of an ideal female parent with high crossability and compatibility [92,93,94,95,96]. In order to improve the crop of buckwheat varieties, many hybridizations between buckwheat species have been attempted, but most of the interspecific crosses have not been successful in obtaining interspecific hybrid progenies as yet, with the exception of the following crosses: autotetraploid *F. esculentum* × tetraploid *F. zuogongense* [92] and tetraploid *F. tataricum* × tetraploid *F. cymosum* [69,97,98,99,100,101]. Among these studies is the research by Krotov and Dranenko [69], who were involved in extensive hybridization of the perennial buckwheat of the *F. cymosum* and another species and produced manmade new species of buckwheat (*F. giganteum*) with white hetero-style flowers (Table 1). Furthermore, the new species was cultivated because of reduced size seeds and much more shell than common and Tartary buckwheat. After interspecific hybridization embryos were rescued, they were inoculated in culture media and cultivated in in vitro conditions. Most part of the biotechnological breeding is bound to in vitro conditions (Figure 2).

## 5. Tissue Culture and Plant Regeneration

During the last 30 and more years, modern biotechnological approaches to the study of the buckwheat genome and breeding in combination with in vitro cultivation have made it possible to obtain modern varieties with more reliable yields. In recent decades, several researchers have studied in detail the efficient methods of somatic cell differentiation in the different buckwheat explants. The first differentiation of five buckwheat plants from cultivated callus, which formed on isolated cotyledons, was obtained by Yamane [102] after 48 months of subculture. All stages of buckwheat explant growth and regeneration in vitro were developed by Srejovic and Neskovic [103]. To regenerate the whole plantlets from the cotyledon fragments, they provided three different media, which were subsequently used on a specific phase of growth.

Plant regeneration via callus formation and subsequent morphogenesis was obtained in many experiments with some modifications of culture media from various explants, such as cotyledon or hypocotyl segments [104,105,106,107,108,109,110,111,112,113,114,115], immature inflorescence [116], and anthers [117,118]. The regenerative ability of the buckwheat explant was significantly influenced by the genotype [119,120]. Somatic embryogenesis is one of the successful options for obtaining regenerants. Gumerova et al. [114,121,122] performed several experiments to study somatic embryogenesis. Indirect somatic embryogenesis was induced via the development of proembryogenic cell complexes, which formed from the hypocotyl explants of 4–5 days old seedlings. Rumyantseva et al. [123,124] established the long-term culture of morphogenic calli of Tartary buckwheat. Kostyukova and Rumyantseva [125] studied the formation of embryoidogenic callus, connected with certain competent cells in procambial and subepidermal layers from cotyledons of immature embryos of Tartary buckwheat. Somatic embryogenesis from hypocotyls of Tartary and common buckwheat inducing callus formation followed by plantlet regeneration with the use of a complex combination of growth regulators has been developed [115,126].

The induction of multiple shoots by direct organogenesis from cotyledons of buckwheat seedlings was obtained by Park and Park [127].

Effective induction of haploids and dihaploids is very important in the rapid acquisition of homozygous lines for the breeding of hybrid varieties, especially common buckwheat. This would make it possible to improve the characteristics that are currently a limiting factor for achieving elite varieties. Basic studies of haploid induction have in the past been carried out by the androgenesis pathway and gynogenesis. The latter is for purposes of determining the possibility of inducing a higher percentage of regenerants with fewer genetic aberrations.

There are few reports on plant regeneration in the anther culture of buckwheat. Adachi et al. [128] produced the first regenerated whole plants in anther culture of the diploid common buckwheat. The regenerants were all diploid. The authors did not exclude the chromosome duplication in plant regenerants, which is a common occurrence in some species [129].

In the experimental androgenesis of Kong et al. [130], regenerants were obtained in all six varieties used. The plants grew well but did not set the seeds. Unfortunately, the number of chromosomes was not counted in this case to confirm the level of ploidy. Bohanec et al. [118] were the first to prove the presence of haploid plants among the regenerants of the diploid common variety. They noted the tendency toward endoduplication because they detected the presence of diploid, triploid, tetraploid, and aneuploid cells. Berbec and Doroszewska [131] induced regeneration in an anther culture of two diploid varieties and one tetraploid. The most regenerants were tetraploids, and no haploids were among the regenerants derived from the diploid varieties; likewise, no diploid regenerants were obtained from the tetraploid variety. The authors assumed that polyploid regenerants must have arisen through the multiplication of the initial chromosome number in microspores or somatic tissues. Yui and Yoshida [132] obtained a number of plantlets regenerated in an anther culture of three diploid varieties. Most of the regenerants were diploid. Wang and Campbell [133] tried to apply the anther culture in the buckwheat breeding process. Among the seven genotypes tested, three were self-pollinated lines developed from interspecific hybridization between common buckwheat and wild *F. homotropicum*, and two genotypes were F1 hybrids. Tetraploid and somatic diploid regenerants were predominant, and no haploid plants were found. The authors concluded that buckwheat is a difficult species in which to produce haploid or dihaploid regenerants via anther cultures.

More success with haploid regenerants is to be expected from isolated haploid microspore cells at the appropriate developmental stage, i.e., late mononuclear to binuclear stage.

The culture of unpollinated ovules (gynogenesis) is less used as an option to obtain haploid plants compared to androgenesis. It has been considered in two studies. One of them dealt with the comparison of responsiveness between anther and ovule cultures, and the other study focused on determining the optimal medium for stable induction and regeneration of haploid plants [118,134]. The protoplast culture technique and regeneration were developed for common [117] and Tartary buckwheat [108] and their fusion [135] for further somatic hybridization, which was later replaced by embryo rescue and genetic transformation for bioactive compound production (Figure 2). For the synthesis and production of pharmaceutically important bioactive compounds, a number of in vitro techniques have been studied, such as in vitro cell line establishment, in vitro cell suspension cultures, *Agrobacterium*-mediated gene transfer, and in vitro hairy root cultivation. Hairy root cultures of common buckwheat have been used to examine the biosynthesis of rutin and other polyphenols in vitro [136,137,138,139] and Tartary buckwheat [140,141,142,143,144].

## 6. Marker Systems for Property Studies

Molecular markers allow the detection of certain properties in plants and thereby more effective and shorter plant breeding programs. Plant breeding using molecular markers has become more accurate and cheaper. In the study of the buckwheat genome, the most commonly included molecular markers are described below (Figure 2).

### 6.1. Random Amplified Polymorphic DNA

Random amplified polymorphic DNA (RAPD) markers are based on the amplification of random DNA segments with single, short (10 nucleotides), and random primers. Fragments of DNA are amplified if two hybridization sites are close enough to each other and in opposite directions. Fragments are detected on agarose gel and are generally scored as dominant markers. RAPD markers are well suited for genetic mapping, DNA fingerprinting, and especially for population genetics [145]. No specific nucleotide sequence information is needed; the method is simple, low-cost, and is not labor-intensive. However, reaction conditions need to be well-defined to obtain reproducible patterns [146].

If RAPD markers of interest are sequenced and longer, more specific primers are designed, we can convert those markers into sequence characterized amplified regions (SCAR) markers which are codominant and can be reproducibly amplified [147].

Among the first DNA markers for agronomically important genes in buckwheat were RAPD markers. Bulked segregant analysis of F2 interspecific hybrids between *F. esculentum* and *F. homotropicum* was used to successfully identify three RAPD markers linked to the self-compatibility gene (*S^h^*) [82,148]. They were then converted into highly reproducible SCAR markers, and one of them (named SCQ7800) was codominant and could be used to distinguish between heterozygous (*S^h^*/*s*) and both homozygous (*S^h^*/*S^h^*) and (*s*/*s*) plants [148].

Tsuji and Ohnishi [149] used RAPD markers to investigate the phylogenetic relationships among cultivated landraces and natural populations of wild subspecies of Tartary buckwheat. The study revealed that the most probable candidate for the original birthplace of cultivated Tartary buckwheat is north-western Yunnan in China (Table 2).

Sharma and Jana [150] used RAPD markers to examine the diversity among 52 landraces and varieties of Tartary buckwheat and one accession of its wild ancestor, *F. tataricum* spp. *potanini*. The study demonstrated the usefulness of the RAPD technique for the characterization of buckwheat genetic resources and the assessment of diversity between species. It also reconfirmed that the origin of cultivated Tartary buckwheat was in Yunnan, as discovered by Tsuji and Ohnishi [149]. Similarly, RAPD was used to detect genetic diversity and relationships among cultivated and wild accessions of Tartary buckwheat and accessions of common buckwheat from the gene bank maintained at the Biotechnical Faculty, University of Ljubljana [151,152].

The assessment of genetic variation in a species is important for the initiation of effective breeding programs because it provides the basis for the selection of desirable genotypes. RAPD markers provide the technical simplicity and speed for the elucidation of inter- and intra-specific variations. Rout and Chrungoo [153] studied genetic variation and phylogenetic relationships in different accessions of Himalayan buckwheat (*F. esculentum*, *F. tataricum* and *F. cymosum*). They used 20 primers, and three of these generated robust and easily interpretable results. The primers used in the study showed a high ability to discriminate between accessions from the same as well as different species of the genus. The dendrogram generated on the basis of RAPD profiles revealed clustering of accessions into three groups that corresponded to species, with various subgroups. An accession identified as *F. himalianum* showed 85-90% similarity with the *F. esculentum* group and should thus be regarded only as a race of *F. esculentum*.

Pan and Chen [154] carried out genetic mapping of common buckwheat using RAPD, STS, seed protein subunits, and morphological markers. A total of 225 plants of the F2 progeny of wild self-fertile *F. esculentum* var. *homotropicum* and diploid heterostylous self-incompatible variety ‘Sobano’ were used for genetic analysis. A total of 99 RAPD and STS markers were used to construct genetic linkage maps. Ten linkage groups were identified (even though buckwheat only has eight chromosome pairs), probably as a result of the absence of linked and shared markers. The authors concluded that the linkage map identified will provide a basis for genetic research into common buckwheat.

Lastly, RAPD markers were used to help confirm successful interspecific hybridization of *F. tataricum* (2n, 4n) with *F. esculentum* (2n, 4n) [155] and *F. esculentum* (2n) with *F. homotropicum* (4n) [156] (Table 1).

### 6.2. Amplified Fragment Length Polymorphism

With the development of the amplified fragment length polymorphism (AFLP) technique, low reproducibility and a need for well-defined reaction conditions of RAPD markers were overcome. The technique is based on four steps: (i) restriction of the DNA with two restriction enzymes and ligation of oligonucleotide adapters, (ii) preamplification with primers having one selective base, (ii) selective amplification with primers having three selective bases, and (iv) gel analysis. The AFLP technique is robust, reliable, and insensitive to the template DNA concentration, and no prior sequence knowledge is needed. The technique produces high marker density and can be used to construct high-density genetic maps [157]. AFLP markers are also useful in studying genetic variation and phylogenetic relationships, the genotyping of individuals and the identification of closely linked DNA markers [158].

Yasui et al. [88] were the first to create a high-density buckwheat genetic map with AFLP markers spanning the whole genome. Twenty primer combinations generated a total of 669 bands, of which 462 were polymorphic and segregated in the F2 population used for mapping. The map of *F. esculentum* had 223 markers covering 548.9 cM. The map of *F. homotropicum* had 211 markers covering 489 cm. In both maps, eight linkage groups were identified that correspond to the eight chromosome pairs. They also successfully mapped three morphological trait genes: distylous self-incompatibility (*S*), shattering habit (*Sht*), and winged seeds (*Wng*). *Sht* and *S* genes were tightly linked (1.3 cM). In a breeding program where *F. homotropicum* is used to transfer the self-compatible trait to *F. esculentum*, rare recombinants that have both self-compatibility and non-shattering habit are desired (Table 2).

Five AFPL markers, tightly linked to the *sht1* non-seed shattering locus, were developed by Matsui et al. [159]. Two of these markers cosegregated with the *sht1* locus without recombination and were then converted to SCAR markers, which were both dominant. Developed markers can be used for MAS in the breeding of self-compatible lines by detecting and removing plants that are homo- or heterozygous for the *Sht1* gene before flowering and thus speeding the development of self-compatible and non-seed shattering lines. Nagano et al. [87] also developed nine AFLP markers linked to the *Sh* allele, and two were converted to SCAR markers and one revealed co-dominance.

Yasui et al. [160] constructed a bacterial artificial chromosome (BAC) library for common buckwheat that included 142,002 clones with an average insert size of approximately 76 kb. To demonstrate the utility of the BAC library, they developed two AFLP markers tightly linked to the *dwE* locus and one marker tightly linked to the *S* locus. One marker linked to the *dwE* locus was successfully converted to the codominant SCAR marker that was used to initiate positional cloning of the causative genes.

Since AFLP markers are able to detect many DNA fragments, they have been used for phylogenetic analysis. Konishi et al. [161] analyzed the genetic relationship among seven cultivated and eight natural populations of wild common buckwheat. Comparing 296 generated AFLP markers, the genetic distance between populations was estimated, and a phylogenetic tree was constructed. The Sanjiang area group was found to be more closely related to cultivated buckwheat, which indicated that the Sanjiang area could be the original birthplace of cultivated common buckwheat. AFLP markers were also used to study the genetic diversity of Tartary buckwheat. Hou et al. [162] analyzed 165 accessions of Tartary buckwheat from four different geographical regions using 20 informative primer pairs. A total of 114 polymorphic loci were detected, and structure analysis showed that the diversity and genetic relationship of Tartary buckwheat correlate to their geographic distribution.

Gupta et al. [163] used AFLP fingerprinting to identify fingerprint profiles unique to high-rutin content accessions of Tartary buckwheat. AFLP analysis successfully grouped high- and low-rutin content accessions into two separate groups. AFLP fingerprinting can therefore be used to identify high-rutin content accessions, which would be useful in breeding programs, for genetic studies and the improvement of buckwheat, for instance, searching for and cloning novel genes/alleles and contributing to higher flavonoid content (Figure 2, Table 2).

**Table 2:** Groups of markers used for genomic studies of the genus *Fagopyrum*.

Year	Marker	Objectives of the Study	Results	Reference
1987	Morphological and allozyme	To analyze linkage relationship between morphological and allozyme marker	30 morphological trait loci were identified, and first common buckwheat linkage map was constructed	[164]
1998; 1999	RAPD, SCAR	To identify RAPD markers linked to the homostylar (*Ho*) gene	3 RAPD markers (one successfully converted to SCAR) linked to the *Ho* gene were developed	[82,148]
2000	RAPD	To study phylogenetic relationship among wild and cultivated Tartary buckwheat	Phylogenetic tree was constructed; north-western Yunnan is most likely the origin of cultivated Tartary buckwheat	[149]
2004	RAPD	Characterization of interspecific hybridization between *F. esculentum* and *F. homotropicum*	RAPD markers were able to successfully determine F1 hybrids between *F. esculentum* and *F. homotropicum*	[156]
2004	AFLP	To perform linkage analysis of *F. esculentum* and *F. homotropicum*	First high-density genetic map with genome-wide AFLP markers was constructed, and three morphological trait genes were mapped	[88]
2005	AFLP	To study genetic relationship among cultivated and wild common buckwheat	Phylogenetic tree was constructed; cultivated common buckwheat probably originated from Sanjiang area	[161]
2012	AFLP	To characterize Tartary buckwheat for rutin content variation	AFLP fingerprinting can be used to identify high rutin content accessions	[163]
2006	SSR	To develop SSR markers for common buckwheat	54 SSR markers were developed, and transferability in closely related species was demonstrated	[165]
2006	SSR, AFLP	To construct linkage map for common buckwheat with SSR and AFLP markers	Female and male linkage map with 12 linkage groups (8 large) was constructed. 19 SSR markers also worked in Tartary buckwheat	[166]

### 6.3. Genome Studies by Next-Generation Sequencing Methods

Yabe et al. [167] applied a novel microarray-based system, and Enoki et al. and Iehisa et al. [168,169] applied linkage analysis and QTL mapping to common buckwheat. Microarray probes were designed based on next-generation sequencing (NGS) data obtained from complexity-reduced genomic DNA and used for the construction of a high-density linkage map and for QTL mapping. A high-density linkage map with multiple markers at a single position (756 loci and 8884 markers) and with an average interval of adjacent markers of 2.12 cM was constructed, which is better than most linkage maps developed to date. Four QTLs for stem length were also mapped. The same group [170] later used the developed marker system to evaluate the potential of genomic selection (GS) [171] in common buckwheat and successfully used the technology to increase yield. Successful application of GS in buckwheat breeding was demonstrated, and the main obstacles that will have to be overcome for the wider use of GS in the breeding of buckwheat were identified (Figure 2).

In 2016, the buckwheat genome was finally sequenced using NGS technology [75]. The 1.2 Gbp-sized draft genome was annotated (>35.000 genes were predicted), and the publicly available Buckwheat Genome DataBase (BGDB; http://buckwheat.kazusa.or.jp, accessed on 5 March 2021) was constructed. To demonstrate the utility of the database, several agronomic useful genes were identified (genes that control flavonoid biosynthesis, a buckwheat allergen gene, and genes for granule-bound starch synthases). The draft genome was used as a reference genome for GBS (genotyping-by-sequencing) analysis to discover and locate the 5.4 Mbp *S*-allelic region where several candidate genes controlling buckwheat heteromorphic self-incompatibility were identified [75]. Ethyl-methane sulphonate (EMS)-induced mutant pools of buckwheat for these genes are now being screened for the development of SC lines with highly valuable agronomical traits, for instance, low amylose and low-allergen lines [85]. Recently, single-molecule real-time (SMRT) long reads were used together with Illumina short reads for de novo assembly of the common buckwheat genome with 39 times better N50 score (~180 kb) [172] than that of [75]. A transcriptome map, based on the newly assembled genome, was constructed and made publicly available on the TraVA database (http://travadb.org/browse/Species=Fesc/, accessed on 10 March 2021).

Chloroplast genome sequences are already available for common [173], Tartary [174,175], and wild [170] buckwheat species. In chloroplast genomes of common buckwheat, many non-synonymous mutations were found in otherwise highly conserved genes encoding photosystem components, and it was hypothesized that this is the result of artificial selection for increased photosynthesis efficiency in the past [173]. The mitochondrial genome was also sequenced recently [173]. Extranuclear genomes were mostly used in phylogenetic studies [173,176] and in assessing the genetic diversity of buckwheat [173].

In 2017, a 0.489 Gbp-sized genome of Tartary buckwheat was sequenced by combining Illumina short reads and SMRT technology long reads. Here, 33,366 genes were annotated on the reference genome, which was used to identify genes involved in rutin biosynthesis (and their transcription factors) and abiotic stress tolerance [175]. The high-quality Tartary buckwheat genome finally enabled more detailed genome-wide identification, expression analysis, and functional studies of many genes, e.g., 20 *ARF* (*Auxin Response Factor*) [177], 47 *GRAS* [178], 134 *AP2*/*E* [179], and 285 *CYP* (*Cytochrome P450*) genes [180].

NGS sequence data can be used to rapidly identify SSR loci, which can then be used to design specific primers for these loci. Based on a genome survey of Tartary buckwheat, 221 primer pairs for identified SSR loci were designed, and 23 polymorphic loci were successfully used to study the genetic diversity of Tartary buckwheat cultivars [181]. Similarly, RNA sequencing of the immature seed transcriptome of common buckwheat was performed to identify 2326 transcripts that contained SSR motifs. For 150 randomly chosen genic-SSRs, primer pairs were designed. A total of 36 primers pairs were polymorphic in 24 common buckwheat accessions, and 141 genic-SSRs were also successfully amplified in Tartary buckwheat [182].

Genetic locus controlling easy dehulling in Tartary buckwheat was identified by combining bulk segregant analysis (BSA) and high-throughput sequencing. The genetic region spans 857.3 kbp and contains 44 non-synonymous and 1 STOP gain SNP, affecting 36 genes [183]. Fukuie et al. [184] used RNA sequencing to identify a candidate gene for easy dehulling in Tartary buckwheat. A 42 bp deletion due to a G → A substitution in an *Arabidopsis thaliana AGAMOUS* ortholog (*FtAG*) was associated with easy dehulling in the so-called ‘rice-type’ varieties. Specific primers for this locus were developed, and this DNA marker could be useful for breeding new rice-type Tartary buckwheat varieties.

Transcriptome analysis has also proven useful in studying genes and transcription factors related to seed size in common buckwheat [185] and salt-stress response in Tartary buckwheat [186].

## 7. Genetic Transformation and Genome Editing

Plant transformations have become one of the tools in plant research and breeding. Many methods for obtaining transgenic plants have been developed and optimized for the main cereal crops, such as maize [187], rice [188], and wheat [189]. In buckwheat, a minor pseudocereal crop, very little research has been done in regard to transformations, even though the first successful *Agrobacterium*-mediated gene transfer was performed as early as 1992 [110]. *Agrobacterium*-mediated transformation is also the only method used in in vitro buckwheat transformations; no other methods (i.e., biolistics, microinjection, viral vectors, etc.) have been reported so far, except for polyethylene glycol (PEG)-mediated transient transformation of protoplasts [190].

### 7.1. In Vitro Transformations

**Figure 3:**
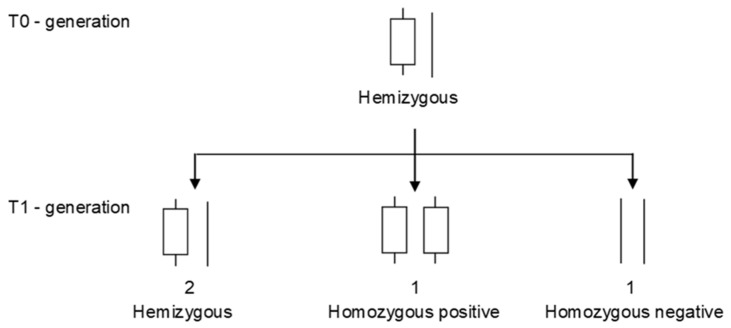
Genetic structure of hemizygous plant T0 and after self-pollination of the resulting progeny T1. Transformed plants regenerated from callus, or after in planta transformation of germinating seedlings, are termed T0 plants. T0 plants are always hemizygous, meaning that there is a copy of the transgene at a novel locus in the plant genome; the DNA gets integrated into one chromosome, but not the homolog at the same locus. T0 plants produce T1 seeds, which in turn develop into T1 plants that carry the transgene in either a hemizygous, homozygous positive, or homozygous negative state in an expected 2:1:1 Mendelian ratio if there is a single copy insertion of the transgene.

The first successful transformation of buckwheat was reported in 1992 [110]. Excised cotyledon fragments were co-cultivated with *A. tumefaciens* (A281 strain harboring the binary vector pGA472 with *nptII* reporter gene for kanamycin resistance) and cultured on regeneration medium. Regenerated shoots were then subjected to kanamycin selection. The transformation was confirmed with *nptII* activity assay and DNA hybridization. Transformed plants were then cross-pollinated, and the seeds plants showed the expected 3:1 segregation (single dominant gene) ratio for kanamycin resistance. Similarly, hypocotyl segments can also be used for generating transgenic common buckwheat (Figure 3) [191].

There has been only one reported in vitro transformation of buckwheat with an agronomically important gene [192]. *A. tumefaciens* was used to transfer the *Arabidopsis* vacuolar Na^+^/H^+^ antiporter gene, conferring salt tolerance. The primary goal of the study was to develop and optimize protocols for callus induction and the transformation and regeneration of transformed plants. The highest transformation frequency was achieved when fresh calli from hypocotyls were precultured for two days, submerged in *A. tumefaciens* suspension for 25 min and then cocultured for 1–2 days. Interestingly, acetosyringone did not significantly increase transformation frequency. The transformation was confirmed by PCR and Southern blotting. Northern blotting and RT-PCR were used to determine expression levels of the inserted gene. Transformed plants were able to grow normally at 200 mM NaCl and also had higher levels of rutin than control plants, thus demonstrating the potential of such transgenic buckwheat for agricultural production in saline soils.

A simple and efficient method for transient gene expression in common buckwheat was recently developed and optimized [190]. The method is based on enzyme-assisted hypocotyl protoplast isolation and PEG-mediated transfection. Isolated protoplasts are mixed with recombinant plasmid in microtiter plates, and PEG/Ca^2+^ solution is added. After vortexing, protoplasts are washed and incubated for 18 h in the dark. The expression can then be triggered by adding a specific inducer. Because the protocol is simple, robust, and efficient (58 ± 5%), it is suitable for high-throughput experiments for functional analysis of buckwheat genes. If regeneration from protoplast could be optimized, this method would be very useful for buckwheat transformations and for genome editing (i.e., CRISPR, TALEN, ZNF), of which no experiments were reported to date.

In all of the studies described above, *A. tumefaciens* was used as a vector for transformation. *A. rhizogenes* was also used for common [138,139] and Tartary buckwheat [140,141] transformations, but efforts in these studies were mostly directed toward using transformed hairy root culture for studying secondary metabolites (mostly rutin) production and regulation [142,143]. The results of such studies at first glance are not directly useful in the breeding of buckwheat, but through gaining a better understanding of the genetics and regulation of secondary metabolism in buckwheat, new varieties with higher antioxidant or rutin content might be developed.

### 7.2. In Planta Transformations

Because in vitro transformation protocols require the use of tissue culture and in vitro regeneration of plants, in planta transformation of common buckwheat with *A. tumefaciens* was developed [193,194]. Apical meristems of 7-cm high seedlings were gently pricked with a needle and inoculated with a suspension of *A. tumefaciens* (LBA4404 harboring pBII21). Inoculated seedlings were kept in the dark for 3 days at 22 °C and then grown to maturity in a growth chamber. Mature plants were allowed to pollinate randomly, and in seeds, transformation efficiency was estimated by germination in the presence of geneticin (36%) and by PCR analysis (70%). This method has several advantages over in vitro transformation methods: it does not require sterile conditions, it does not involve tissue culture (difficult regeneration and somaclonal variations), transformation efficiency is high, and a large number of transgenic plants can be obtained in a short period [193]. This protocol was then used to study the function of a rice MADS-box gene (accession No. (DDBJ), AB003325) in common buckwheat. Plants transformed with a sense orientation of a cDNA of a MADS-box gene were stimulated in branching, and plants transformed with an antisense orientation of the same gene were inhibited in both branching and growth. The study showed the utility of the developed in planta transformation method for studying gene functions in buckwheat and for developing new agronomically important transgenic lines of buckwheat [195]. Two other in planta methods for transient gene expression were developed by Bratić et al. [196]. Parameters for vacuum infiltration and for infiltration by syringe were optimized, such as *Agrobacterium* culture density, vacuum conditions, and leaf maturity. Under optimized conditions, the vacuum infiltration method was much more efficient, as the GUS activity (reporter gene) was 57.3 times higher than that for infiltration by syringe. The only transformation of Tartary buckwheat with *A. tumefaciens* was performed by Chawla et al. [197]. Imbibed seeds were co-cultivated with *A. tumefaciens* (GV3101 harboring CAMBIA 1301) for 1 h, and seeds were then germinated. Transformation was confirmed in leaves of young seedlings by histochemical detection of β-glucuronidase (36%) and by PCR analysis (22.72%).

### 7.3. Genome Editing

New findings in molecular genetics allow for breeding with newer methods of genome editing at the level of individual nucleotides or a group of nucleotides. Clustered regularly interspaced short polindromic repeats/CRISPR associated protein 9 (CRISPR/Cas9)-based genome editing is a ground-breaking technology in the field of functional genomics [198,199,200]. Precise genome editing for well-defined agronomic traits with the CRISPR/Cas9 approach and other methods transcriptional activator-like effector nucleases (TALEN), zinc-finger nucleases (ZNF) could accelerate the development of buckwheat varieties with improved economic traits, as in the case of grapevine, apple, rice, tomato, banana, maize, wheat, etc. [201,202,203,204,205]. These approaches, although not yet tested in buckwheat, have great potential in buckwheat breeding. However, rapid advancements in buckwheat transformation and genome editing provide the opportunity for the development of high-quality, sustainable nutraceutical products through these technologies in the near future. The CRISPR-edited buckwheat products will be expected to have reduced antinutritional factors and enhanced protein, essential amino acids, and bioactive compounds compared to commercial cultivars [206]. The transformation and genome editing of common buckwheat and Tartary buckwheat have a restriction in the application. In Slovenia and in several other European countries (for example, Austria, Czech Republic, and Italy), common buckwheat and Tartary buckwheat are grown as ecological crops. According to the demand for ecological crops, it is in such crops that the use of transgenic varieties is not allowed. Furthermore, transgenic cultivars should not be cultivated close to ecological crops to prevent pollination of ecologically grown plants with the pollen of transgenic plants. However, for research purposes, there are no such limitations if plants are grown in isolation.

## 8. Conclusions

Buckwheat as a functional food plays an important role in human nutrition. It is characterized by a short growing season and modest nutrient needs. Therefore, it is widespread in almost all production areas, even in regions with higher altitudes. Currently, breeding programs do not result in high-yielding varieties with a stable yield, as in other leading cultivated plants. With the development of molecular genetics, genomics, and progress in interspecific hybridization between genetically distant species of buckwheat and rescuing immature embryos in tissue culture, the chances of successful breeding are going to increase. These newer breeding methods, such as precise genome editing of genes through the CRISPR, TALEN, or ZNF, which have not yet been used in buckwheat, offer the possibility of obtaining varieties with improved agronomic traits faster and more efficiently.

## Data Availability

Not applicable.
